# HTLV-1-host interactions on the development of adult T cell leukemia/lymphoma: virus and host gene expressions

**DOI:** 10.1186/s12885-018-5209-5

**Published:** 2018-12-22

**Authors:** Hanieh Tarokhian, Hossein Rahimi, Arman Mosavat, Abbas Shirdel, Houshang Rafatpanah, Mohammad Mehdi Akbarin, Alireza Bari, Samaneh Ramezani, Seyed Abdolrahim Rezaee

**Affiliations:** 1 Department of Immunology, School of Medicine, Kermanshah University of Medical Sciences, Kermanshah, Iran; 2 Hematology and Oncology Department, Faculty of Medicine, Mashhad University of Medical Sciences, Mashhad, Iran; 3 Blood Borne Infections Research Center, Academic Center for Education, Culture and Research (ACECR), Razavi Khorasan, Mashhad, Iran; 4 Immunology Research center, Inflammation and Inflammatory Diseases Division, Mashhad University of Medical Sciences, Azadi-Square, Medical Campus, Mashhad, Zip code: 9177948564 Iran; 5 Student Research Committee, Faculty of Medicine, Mashhad University of Medical Sciences, Mashhad, Iran

**Keywords:** HTLV-1-proviral load, HBZ, AKT1, BAD, FOXP3, RORγt, IFNλ3

## Abstract

**Background:**

Adult T-cell leukemia/lymphoma (ATLL) is a lymphoproliferative disorder of HTLV-1-host interactions in infected TCD4+ cells. In this study, the HTLV-1 proviral load (PVL) and HBZ as viral elements and *AKT1*, *BAD*, *FOXP3*, *RORγt* and *IFNλ3* as the host factors were investigated.

**Methods:**

The study was conducted in ATLLs, HTLV-1-associated myelopathy/tropical spastic paraparesis patients (HAM/TSPs) and HTLV-1-asympthomatic carriers (ACs). The DNA and mRNA from peripheral blood mononuclear cells were extracted for gene expression assessments via qRT-PCR, TaqMan assay, and then confirmed by western blotting.

**Results:**

As it was expected, the HTLV-1-PVL were higher in ATLLs than ACs (*P* = 0.002) and HAM/TSP (*P* = 0.041). The *HBZ* expression in ATLL (101.76 ± 61.3) was radically higher than in ACs (0.12 ± 0.05) and HAM/TSP (0.01 ± 0.1) (*P* = 0.001). Furthermore, the *AKT1* expression in ATLLs (13.52 ± 4.78) was higher than ACs (1.17 ± 0.27) (*P* = 0.05) and HAM/TSPs (0.72 ± 0.49) (*P* = 0.008). However, *BAD* expression in ATLL was slightly higher than ACs and HAM/TSPs and not significant. The *FOXP3* in ATLLs (41.02 ± 24.2) was more than ACs (1.44 ± 1) (*P* = 0.007) and HAM/TSP (0.45 ± 0.15) (*P* = 0.01). However, *RORγt* in ATLLs (27.43 ± 14.8) was higher than ACs (1.05 ± 0.32) (*P* = 0.02) but not HAM/TSPs. Finally, the *IFNλ3* expression between ATLLs (31.92 ± 26.02) and ACs (1.46 ± 0.63) (*P* = 0.01) and ACs and HAM/TSPs (680.62 ± 674.6) (*P* = 0.02) were statistically different, but not between ATLLs and HAM/TSPs.

**Conclusions:**

The present and our previous study demonstrated that HTLV-1-PVL and *HBZ* and host *AKT1* and *Rad 51* are novel candidates for molecular targeting therapy of ATLL. However, high level of RORγt may inhibit Th1 response and complicated in ATLL progressions.

## Background

The human lymphotropic virus type 1 (HTLV-1) is a member of the *Retroviridea* family, which infects about 10–20 million people in the world. Although, after a long period of time, a small percentage (3–5%) of infected individuals will progress to HTLV-associated myelopathy-tropical spastic paraparesis (HAM/TSP) or Adult T-cell leukaemia/lymphoma (ATLL), the large number of infected carriers remains asymptomatic [1].

The HTLV-1 endemic areas include, Southwestern Japan, the Caribbean basin, Central Africa, the Melanesian Islands, South America and the Middle East [2]. In Iran, at least in four provinces, including; Razavi Khorasan, Northern Khorasan, Golestan and East Azarbaijan HTLV-1 is endemic [3]. Furthermore, HTLV-1- associated diseases have been reported from some other provinces such as Alborz, Tehran and Fars [4].

The HTLV-1 activity and infectivity in asymptomatic carriers and HTLV-1 associated diseases is quantified by the number of infected peripheral blood mononuclear cells (PBMCs), which expressed as the proviral load (PVL) [5]. The increasing amount of PVL results in more expressions of viral genes, such as *Tax* and HTLV-1 bZIP factor (*HBZ*). These regulatory proteins have a close interaction through mutual transcriptional regulation interference in the infected T cells. HBZ as regulatory protein through inhibition of Tax can help to evade host immune responses. Therefore, the over-expression of *HBZ* is implicated on the development of malignancy in the late stages of cell transformation in HTLV-1-infected cells [6]. Moreover, previous studies have reported a correlation between *HBZ* mRNA expression and HAM/TSP disease severity [7, 8].

The Tax and HBZ may exert their oncogenic and pathogenicity effects by changing the outcome of signalling pathways, such as NFκB and phosphoinositide 3-kinase (PI3K), and apoptotic event [8, 9]. AKT or protein kinase B (PKB) is a primary mediator of the PI3K signalling pathway. AKT and other kinases in the PI3K pathway have some substrates that contribute to cell transformation, results in malignancy. AKT can inhibit apoptosis through phosphorylation and the inhibition of pro-apoptotic mediators such as Bcl-2-associated death promoter (BAD) and caspase-9 to contribute to the maintenance of the virus latent state and may facilitate transformation of infected cells [9, 10].

Likewise, BAD and BAX are members of the BCL-2 family proteins and are the main molecules in regulating cell survival and apoptosis [11]. HTLV-1 proteins modulate the levels of BCL-2 family members in favour of virus dissemination [12], and AKT inhibits the BAD activity. BAD as a pro-apoptotic agent is a target of AKT in such circumstances, and helps infected cells to escape cell death [9].

Interferons (types I, II, and III) are the most important antiviral agents in innate and adaptive immune responses. Type III IFNs, includes 3 subtypes, IFN-λ1, λ2 and λ3, known as interleukin-29 (IL-29), IL-28A, and IL-28B, respectively [13]. IFN-λ is known to inhibit replication in a range of viruses, including, hepatitis C and B virus (HCV and HBV), influenza virus, rotavirus, herpes simplex virus-1 and 2, encephalomyocarditis virus, vesicular stomatitis virus, cytomegalovirus, and West Nile virus [14]. This class of IFNs also can induced apoptosis and has direct growth inhibitory action, therefore, has direct antitumor effects and can augment major histocompatibility complex (MHC) class I antigens expression which may stimulate Th1 responses which increases antigen presenting in favor of anti-viral and anti-tumor activities of cytotoxic T cell and anti-angiogenesis [13].

To lower the host immune pressure, *HBZ* overexpresses to suppress the expression of *Tax* as a virus activator and dominated antigen to escape the immune response. Therefore, the persistence and pathogenesis of HTLV-1 depends on the host immune response such as Th1, cytotoxic T lymphocytes (CTLs), natural killer cells (NK-cells) and Th17 [15]. Regulatory T cells (Tregs) that express the forkhead box P3 (*FOXP3*) transcription factor, play a critical role in the maintenance of the immune system homeostasis. An immunosuppressive micro-environment enables HTLV-1-infected cells to escape the host immune responses. Besides, the most HTLV-1-infected cells have been suggested to be the Treg lymphocytes. Many reports have been conducted on the role of Tregs in HTLV-1-associated disease progression [16]. Moreover, viral replication can be enhanced by proinflammatory responses of Th17. In case of HTLV-1 associated diseases the roles of Treg and Th17 cells differ depending on the infection stage and host immune status [17–19]. In the context of IFNs and transcription factors, it is better to elucidate the role of these central arms of immune responses through their main transcription factors FOXP3 and RAR-related orphan nuclear receptor (RORγt).

In the present study, the host-virus interactions in the manifestation of ATLL and HAM/TSP were investigated by assessment of HTLV-1-PVL and *HBZ* as viral elements, *AKT1* as a regulator of the cell cycle progression and cell survival, *BAD* as proapoptetic agent and finally *RORγt*, *FOXP3* genes as the main transcription factor in Th17 and Treg cells, and *IFN-λ3* as an antiviral agent in the innate immune responses.

## Methods

### Subjects and study setting

In this cross-sectional study subjects consisted of 18 ATLL, 10 HAM/TSP newly diagnosed patients, and 18 HTLV-1 ACs from the hematology/oncology and neurology wards of Ghaem referral university Hospital, Mashhad, Iran, between May-2014 and November-2015. Diseases, ATLL and HAM/TSP, in patients were confirmed by two neurologist and oncologist. All participants had a seropositive test for HTLV-1 (ELISA, Dia.Pro, Italy) which infection was confirmed by PCR for *Tax* and *LTR* DNA fragments of provirus.

### RNA and protein extraction, cDNA synthesis

The PBMCs were isolated from whole-blood by Ficoll density gradient (Cederline, Ontario). Total RNA and protein extraction from PBMCs was isolated from TriPure (Roche Diagnostics, Switzerland) treated samples according to the manufacturer’s instructions. The extracted RNA was then reverse-transcribed to cDNA by the AccuPower ®RT PreMix cDNA synthesis kit (Bioneer, Korea). The extracted protein was kept at − 70 °C for subsequent western blotting analysis.

### Oligonucleotide design and gene expression assessments

Primers and probes were designed by Beacon Designer software (PREMIER Biosoft, USA, version 7). Table 1 shows the nucleotide sequence of primers and probes. Real-time qRT-PCR was carried out on the cDNA samples using two standard curve techniques with a Rotor-Gene Q 6000 Machine (Qiagen, Germany). The test was performed with the Universal Master Mix (Takara; Otsu Shiga) for *HBZ*, *AKT1*, *BAD*, *FOXP3*, *RORγt*, *IFNλ3* and the cellular reference gene beta-2 microglobulin (β2M) using the TaqMan method. For assessment of gene expression five standards were prepared using five folds serial dilution of a concentrated sample for the gene of interest and reference gene. Then, standard curves were generated for the target and reference genes, and the data were analyzed using the relative method by Rotor Gene 6000 software (Qiagen, Germany). The relative quantity of the interested gene was normalized to the relative quantity of β2M as the reference gene and expressed as the expression index. Briefly, the relative *AKT1*, *BAD*, *FOXP3*, *RORγt*, *IFNλ3* and *HBZ* expression levels for each sample were calculated by an equation of: normalized Index = copy number of gene of interest (*AKT1*, *BAD*, *FOXP3*, *RORγt*, *IFNλ3* and *HBZ*)/copy number of reference gene (β2M).

**Table 1:** The sequence of primers and probes used for genes expression

Targeted gene	Sequence 5’***→***3’	Purpose	Product length (bp)
*AKT1*	5’-GTGTCAGCCCTGGACTACC-3′ 5’-CAGCCCGAAGTCTGTGATCTTA-3’ FAM-TCCTTGTCCAGCATGAGGTTCTCCAGC-BHQ	Forward Reverse Probe	114
*BAD*	5’-GACGAGTTTGTGGACTCCTTTAAG-3′ 5’-CCTGCCCAAGTTCCGATCC-3’ FAM-TCCTCGCCCGAAGAGCGCGG-BHQ	Forward Reverse Probe	129
*RORγt*	5’-GCTAGGTGCAGAGCTTCAGG-3′ 5’-TGTTCTCATGACTGAGCCTTGG-3’ FAM-CCTTGGCTCCCTGTCCTTCTCAGCA-BHQ	Forward Reverse Probe	145
*FOXP3*	5′- ACTACTTCAAGTTCCACAACATGC -3′ 5′- GAGTGTCCGCTGCTTCTCTG-3’ FAM- TCACCTACGCCACGTTCATCCGCT -BHQ	Forward Reverse Probe	95
*IFNλ3*	5’-GTGGCTTTGGAGGCTGAG-3′ 5’-GCTGGTCCAAGACATCCC-3’ FAM-TTCTGGAGGCCACCGCTGAC-BHQ	Forward Reverse Probe	91
*HBZ*	5’-AGAACGCGACTCAACCGG-3′ 5’-TGACACAGGCAAGCATCGA-3’ FAM-TGGATGGCGGCCTCAGGGCT-BHQ	Forward Reverse Probe	133
β2M	5’-TTGTCTTTCAGCAAGGACTGG-3′ 5’-CCACTTAACTATCTTGGGCTGTG-3’ FAM-TCACATGGTTCACACGGCAGGCAT-BHQ	Forward Reverse Probe	127

### HTLV-1 proviral load measurement

HTLV-1 PVL was assessed on extracted DNA from PBMCs and an absolute real-time PCR was performed using a commercial quantification kit (Novin Gene, Iran) by a Rotorgen real-time PCR machine. The HTLV-1 copy number was reported as an actual amount of cellular DNA by using quantification of the albumin gene as the reference gene. HTLV-1 and albumin DNA concentrations were calculated from two 5-point standard curves. The normalized value of the PVL was calculated as the ratio of (HTLV-1 proviral DNA copies number/albumin DNA copies number/ 2) × 10^4^ and expressed as the number of HTLV-1 proviruses per 10^4^ PBMCs.

### Western blotting analysis

Protein expression was confirmed by western blotting for *AKT1* and *RORγt* genes. Shortly, the proteins were separated on 12% polyacrylamide gel electrophoresis (SDS-PAGE) performance (Bio-Rad, USA), and then the proteins were transferred onto polyvinylidene fluoride (PVDF) membrane (GE Healthcare, USA). After electrotransfring, PVDF membranes were blocked by BSA 2% (overnight at 4 °C). For identification of AKT1 or RORγt protein, membranes were incubated with mouse IgG monoclonal antibody to human beta Actin protein (Abcam, UK) as reference protein at a dilution of 1:500 and mouse IgG monoclonal antibody to human AKT1 (Abcam, UK) or RORγt protein (BD Pharmingen, USA) for 1 h at room temperature. Thereafter, PVDF membranes were incubated for 1 h at room temperature with goat anti-mouse IgG-HRP antibody (Santa Cruz, USA) as secondary antibody at a dilution of 1:10,000. Finally, the specific AKT1 and RORγt proteins were detected by enhanced chemiluminescence detection system (Amersham ECL; GE Healthcare, USA).

### Statistical analysis

pt?>Statistical analysis was performed using SPSS software ver.16.0 (SPSS, Chicago, IL). The results have been shown as mean ± standard error of mean (SEM). Nonparametric statistical tests including Kruskal Wallis (one-way ANOVA) analysis for comparing more than two-independent samples or Mann-Whitney U test for two-independent samples were used for statistical analysis of data. Correlation analysis was performed using Spearman’s test to detect the association between the variables. The *P*-value was considered statistically significant if *P* ≤ 0.05.

## Results

### Studied groups

The study investigated 18 seropositive ATLL patients (11 women and 7 men; mean age 48.64 ± 5.94 years), 10 seropositive HAM/TSP patients (6 women and 4 men; mean age 43.86 ± 8.33 years) and 18 seropositive HTLV-1 ACs (12 women and 6 men; mean age 42.92 ± 6.46 years). There was no significant difference between age and gender in the HTLV-1 ACs, ATLLs and HAM/TSP patients. Place of birth for 85% of the ATLL subjects were Razavi Khorasan province, including 60% in Mashhad, 15% in Neyshabur, 10% in Sabzevar and 15% remaining from other provinces.

### Clinical finding in ATLL group

Among the patients, 61% had lymphadenopathy, 21% had immunodeficiency disorders, and 18% had skin lesion. Two patients had lymphadenopathy and skin lesion, simultaneously, and one patient had immunodeficiency and skin lesion. Note that none of the patients showed three clinical symptoms simultaneously.

### Proviral load

**Fig. 1:**
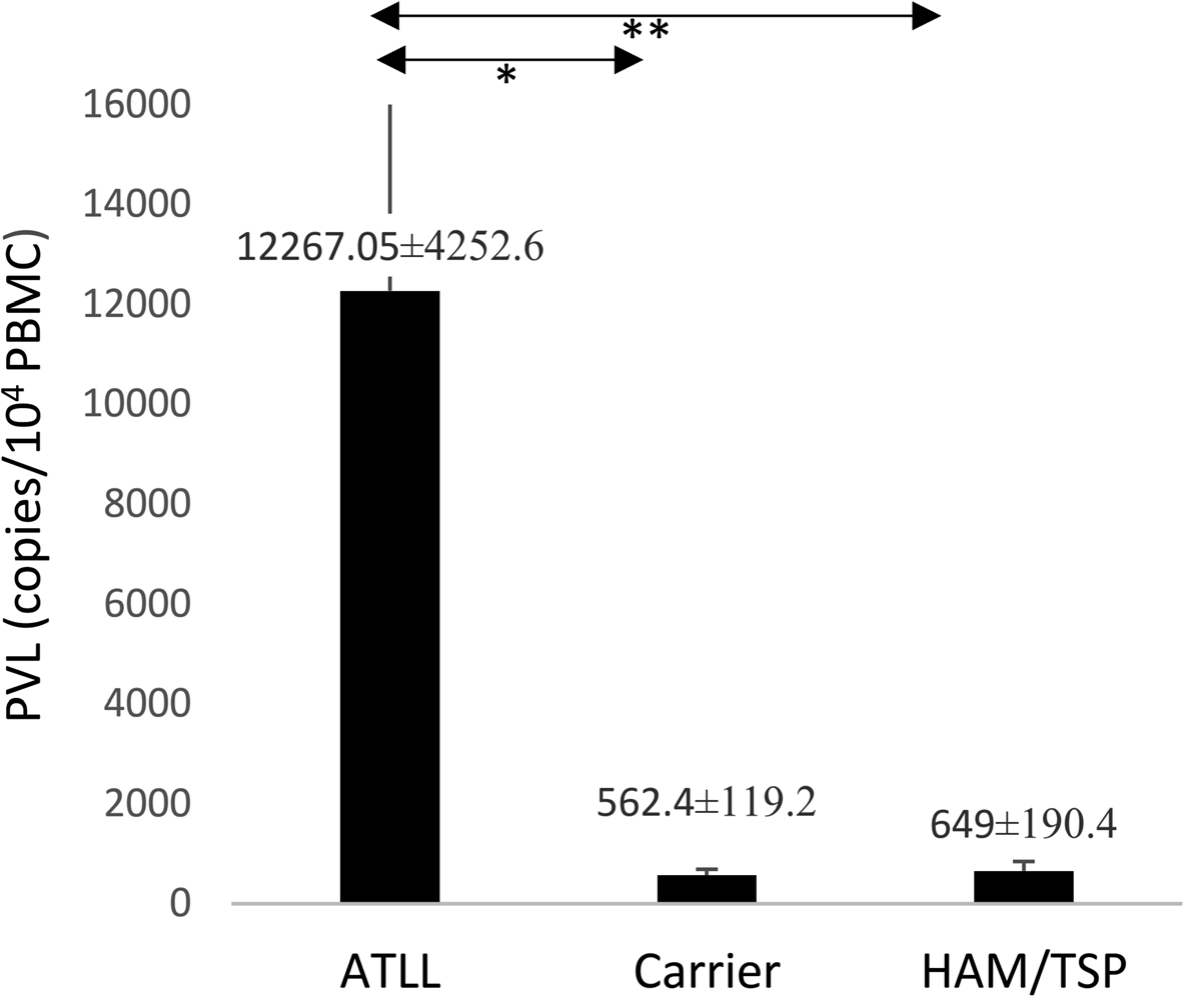
HTLV-I- proviral load in ATLL, ACs, and HAM/TSP groups. The PVL in ATLL patients was significantly higher than in ACs (*P* = 0.003, Mann–Whitney U test). The PVL between ATLL and HAM/TSP patients was statistically different (*P* = 0.041, Mann–Whitney U test). No significant difference was found between ACs and HAM/TSP. *P < 0.05, **P < 0.01. PVL, proviral load; ATLL, adult T-cell leukemia/lymphoma; ACs, asympthomatic carriers; HAM/TSP, HTLV-1-associated myelopathy/tropical spastic paraparesis

Quantitative real-time PCR was performed on the DNA extract of PBMCs. The mean PVL was 11,431.70 ± 3774.3 copies /10^4^ PBMCs in ATLL patients, 562.4 ± 119.2 copies/10^4^ PBMCs in ACs and 649 ± 190.4 copies /10^4^ PBMCs in HAM/TSP patients. The PVL in ATLL patients was significantly higher than that in ACs (*P* = 0.002). The difference of PVL between ATLL and HAM/TSP patients was statistically significant (*P* = 0.041). No significant difference was found between ACs and HAM/TSP patients (Fig. 1).

### HBZ gene expression

**Fig. 2:**
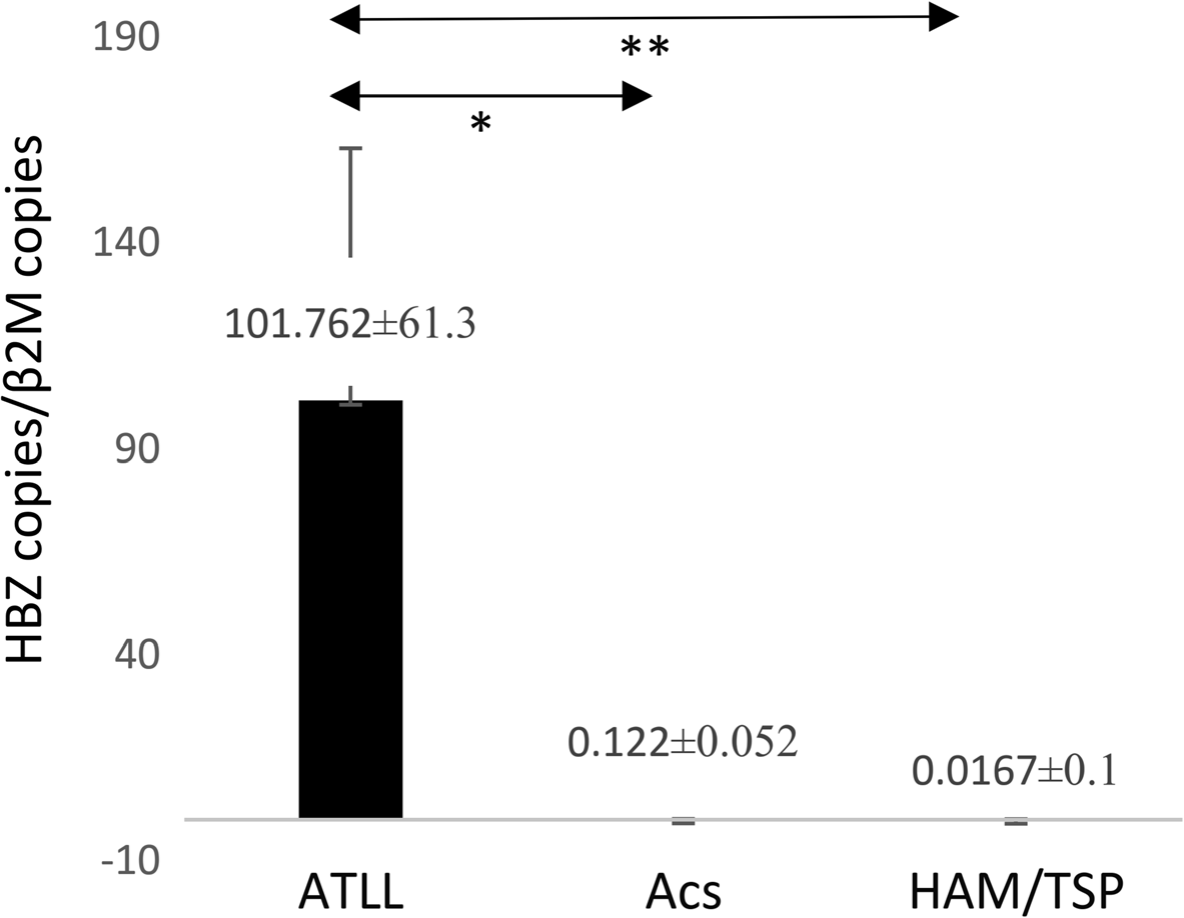
*HBZ* gene expression in ATLL, ACs, and HAM/TSP groups. HBZ gene expression in the ATLL was significantly higher than in ACs (*P* = 0.000, Mann–Whitney U test). The *HBZ* between ATLL and HAM/TSP patients was statistically different (*P* = 0.000, Mann–Whitney U test). No significant difference was found between ACs and HAM/TSP patients. *P < 0.05, **P < 0.01. HBZ, HTLV-1 bZIP factor; adult T-cell leukemia/lymphoma; ACs, asympthomatic carriers; HAM/TSP, HTLV-1-associated myelopathy/tropical spastic paraparesis

The mean *HBZ* gene expression in the ATLL group was strongly higher than that in ACs (101.76 ± 61.3 vs. 0.12 ± 0.05, respectively; *P* = 0.000). The expression of this viral factor was considerably higher in the ATLL group than in the HAM/TSP group (101.76 ± 61.3 vs. 0.01 ± 0.1 respectively; *P* = 0.001). No significant difference was found between ACs and the HAM/TSP group (Fig. 2).

### AKT1 gene expression

**Fig. 3:**
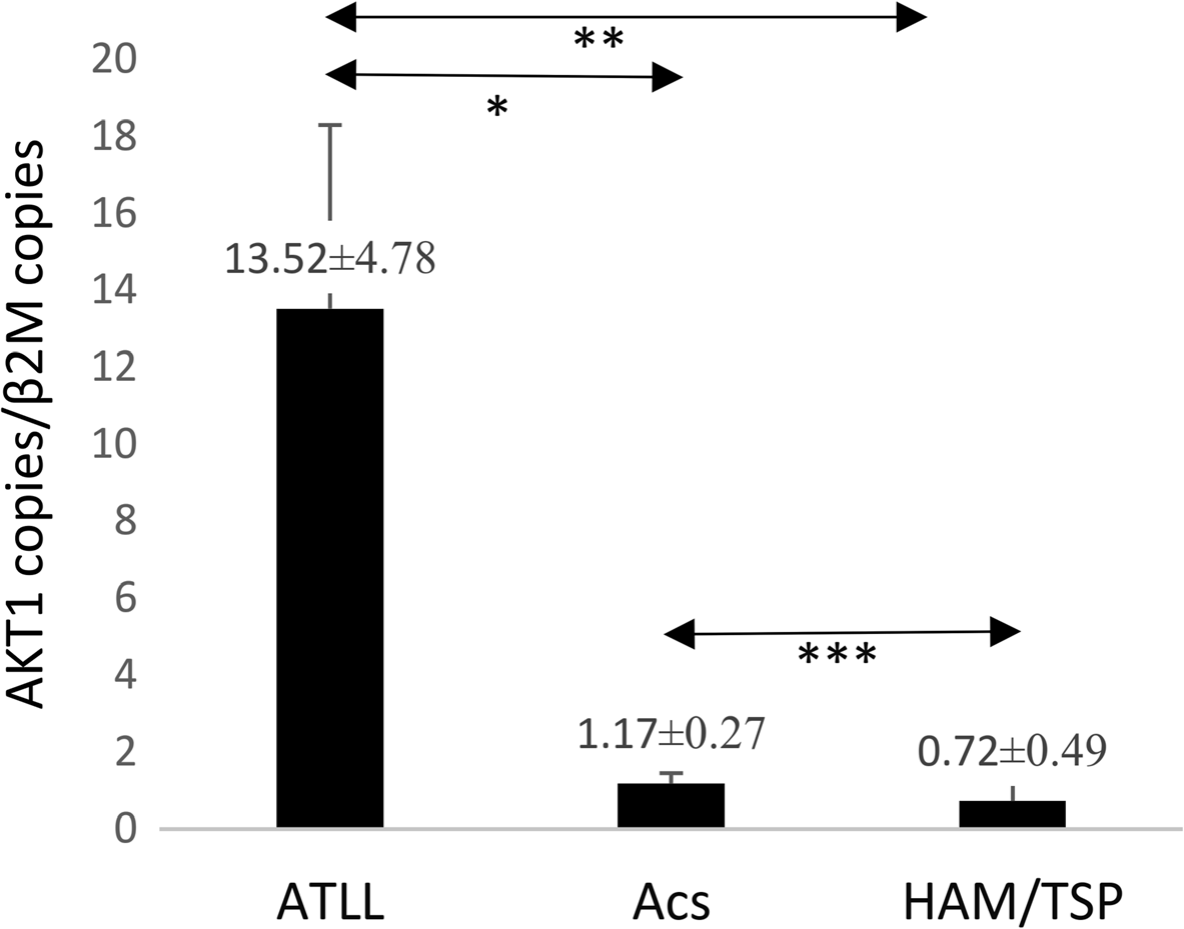
*AKT1* gene expression in ATLL, ACs, and HAM/TSP groups. The expression of *AKT1* in the ATLL patients was higher than in ACs (*P* = 0.05, Mann–Whitney U test) and HAM/TSP patients (*P* = 0.008, Mann–Whitney U test). A significant difference was found in the *AKT1* gene expression between HAM/TSP patients and ACs. *P < 0.05, **P < 0.01, ***P < 0.001. AKT1, serine/threonine kinase 1; adult T-cell leukemia/lymphoma; ACs, asympthomatic carriers; HAM/TSP, HTLV-1-associated myelopathy/tropical spastic paraparesis

The mean expression index of *AKT1* in the ATLLs, ACs, and HAM/TSPs, was 13.52 ± 4.78, 1.17 ± 0.27 and 0.72 ± 0.49, respectively. The *AKT1*expression in ATLL patients was about 12–18 times more than that in ACs (*P* = 0.05) and HAM/TSP patients (*P* = 0.008). A significant difference was found in the *AKT1* gene expression between the HAM/TSP patients and ACs (*P* = 0.027) (Fig. 3).

### BAD gene expression

**Fig. 4:**
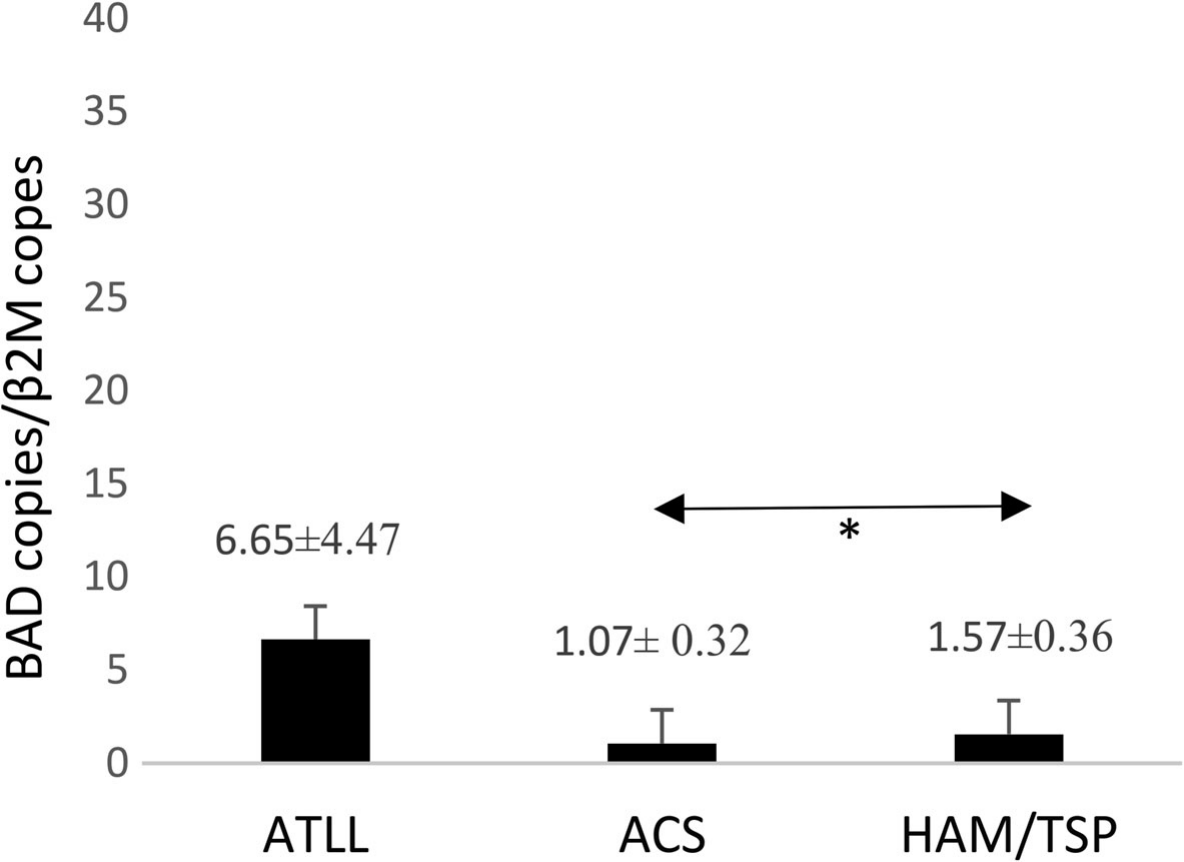
*BAD* gene expression in ATLL, ACs, and HAM/TSP groups. There was significant difference in the expression of *BAD* between ACs and HAM/TSP patients (*P* = 0.027, Mann–Whitney U test). No significant difference was found in the *BAD* gene expression among ATLL patients, ACs and HAM/TSP patients. *P < 0.05. BAD, Bcl-2-associated death promoter; adult T-cell leukemia/lymphoma; ACs, asympthomatic carriers; HAM/TSP, HTLV-1-associated myelopathy/tropical spastic paraparesis

No significant difference was found in the *BAD* gene expression among ATLL patients (6.65 ± 4.47), ACs (1.07 ± 0.32) and HAM/TSP patients (1.57 ± 0.36). However, a significant difference in the *BAD* expression was found between ACs and HAM/TSP patients (*P* = 0.027) (Fig. 4).

### FOXP3 gene expression

**Fig. 5:**
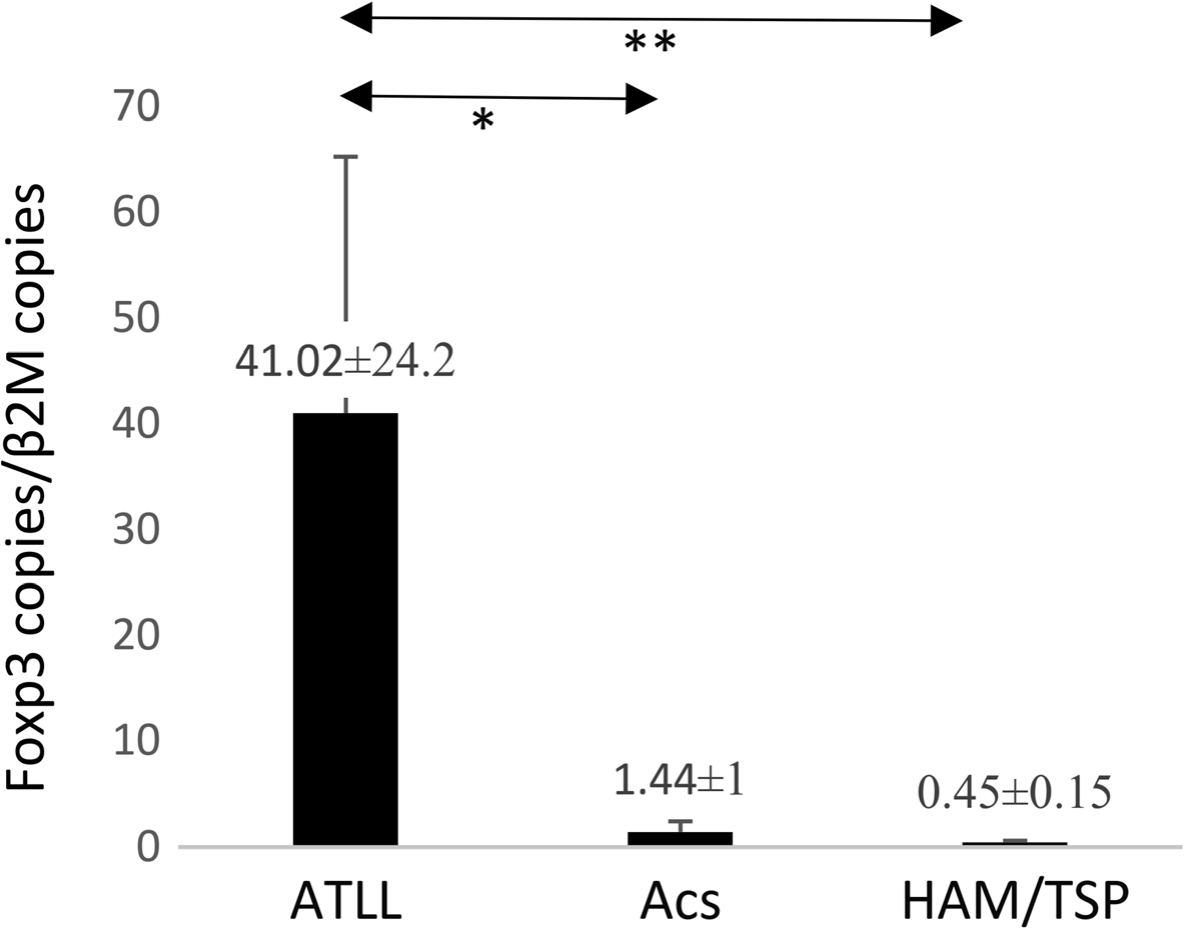
*FOXP3* gene expression in ATLL, ACs, and HAM/TSP groups. The *FOXP3* gene expression in ATLL was significantly higher than in ACs (*P* = 0.007, Mann–Whitney U test) and HAM/TSP patients (*P* = 0.01 Mann–Whitney U test). No significant difference was found between ACs and HAM/TSP group. *P < 0.05, **P < 0.01. FOXP3, forkhead box P3; adult T-cell leukemia/lymphoma; ACs, asympthomatic carriers; HAM/TSP, HTLV-1-associated myelopathy/tropical spastic paraparesis

The mean *FOXP3* gene expression in ATLL was around 28 times more than that in ACs (41.02 ± 24.2 vs. 1.44 ± 1; *P* = 0.007) and 91 times more than HAM/TSP patients (41.02 ± 24.2 vs. 0.45 ± 0.15; *P* = 0.01). No significant difference was found between ACs and the HAM/TSP group (Fig. 5).

### RORγt gene expression

**Fig. 6:**
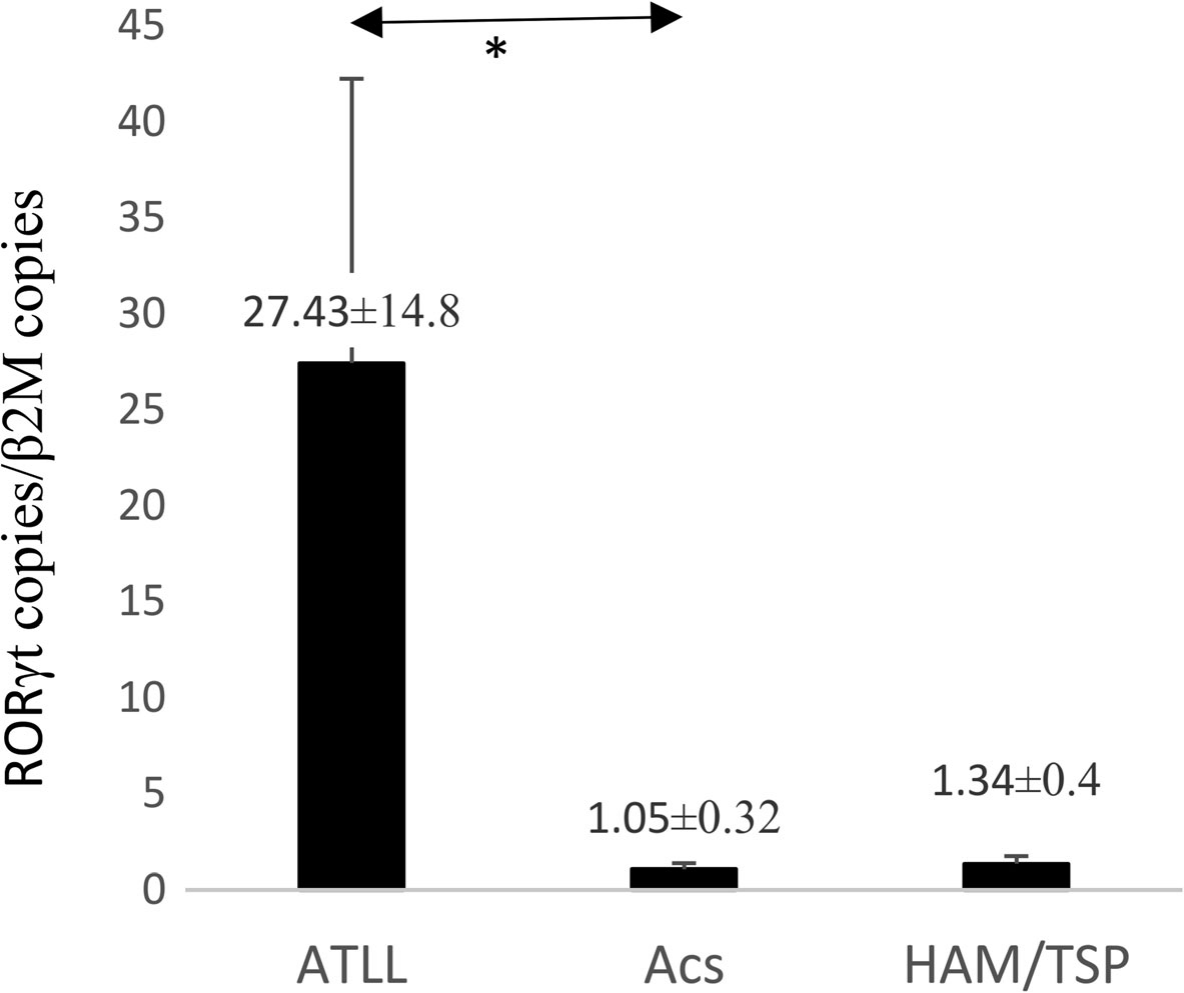
*RORγt* gene expression in in ATLL, ACs, and HAM/TSP groups. The mean *RORγt* gene expression in ATLL patients was higher than that in ACs (*P* = 0.02, Mann–Whitney U test). No significant difference was found in the expression of *RORγt* between ATLL and HAM/TSP patients, also between ACs and HAM/TSP patients. *P < 0.05. RORγt, RAR-related orphan nuclear receptor; adult T-cell leukemia/lymphoma; ACs, asympthomatic carriers; HAM/TSP, HTLV-1-associated myelopathy/tropical spastic paraparesis

The mean expression index of *RORγt* in the ATLL group, ACs, and HAM/TSP group was 27.43 ± 14.8, 1.05 ± 0.32 and 1.34 ± 0.4, respectively. The mean *RORγt* gene expression in ATLL patients was higher than that in ACs (*P* = 0.02). No significant difference was found in the *RORγt* expression between ATLL and HAM/TSP patients, or between ACs and HAM/TSP patients (Fig. 6).

### IFNλ3 gene expression

**Fig. 7:**
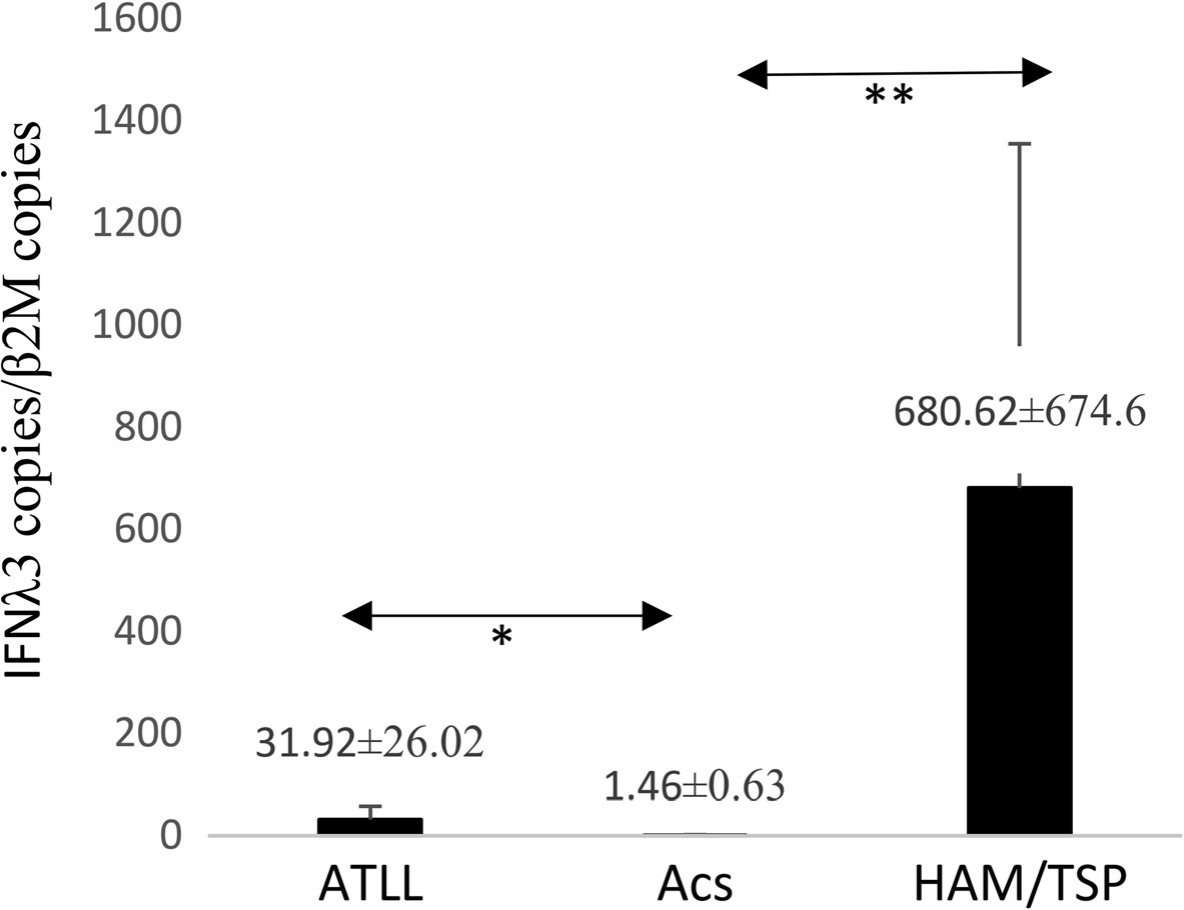
*IFNλ3* gene expression in ATLL, ACs, and HAM/TSP groups. The mean expression index of *IFNλ3* in ATLL patients was higher than that in ACs (*P* = 0.01, Mann–Whitney U test). Moreover was observed significant difference between ACs and HAM/TSP patients (*P* = 0.02, Mann–Whitney U test). However no significant difference was found between ATLL and HAM/TSP patients in the expression of *IFNλ3*. *P < 0.05, **P < 0.01. IFN λ3, Interferon λ3; adult T-cell leukemia/lymphoma; ACs, asympthomatic carriers; HAM/TSP, HTLV-1-associated myelopathy/tropical spastic paraparesis

The mean expression index of *IFNλ3* in ATLL patients was higher than that in ACs (31.92 ± 26.02 vs. 1.46 ± 0.63, *P* = 0.01). Moreover, a significant difference was observed between ACs and HAM/TSP patients (1.46 ± 0.63 vs. 680.62 ± 674.6, *P* = 0.02). However, no significant difference was found between ATLL and HAM/TSP patients in the *IFNλ3* expression (Fig. 7).

### Protein expression levels

**Fig. 8:**
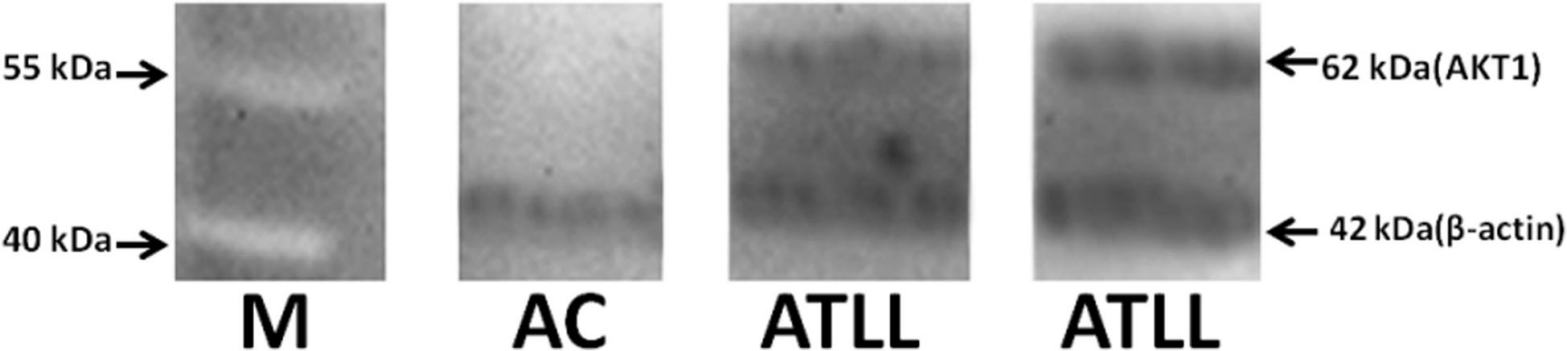
AKT1expression analysis in ATLL and AC subjects. The expression of AKT1 (62 kD) and beta actin (42 kD) were analysed in equal protein amounts of PBMCs lysate of the subjects by western blot with anti-AKT1 and anti-beta-actin horseradish peroxides. AKT1, serine/threonine kinase 1; ATLL, adult T-cell leukemia/lymphoma; ACs, asympthomatic carriers; M, molecular size marker (kDa)

**Fig. 9:**
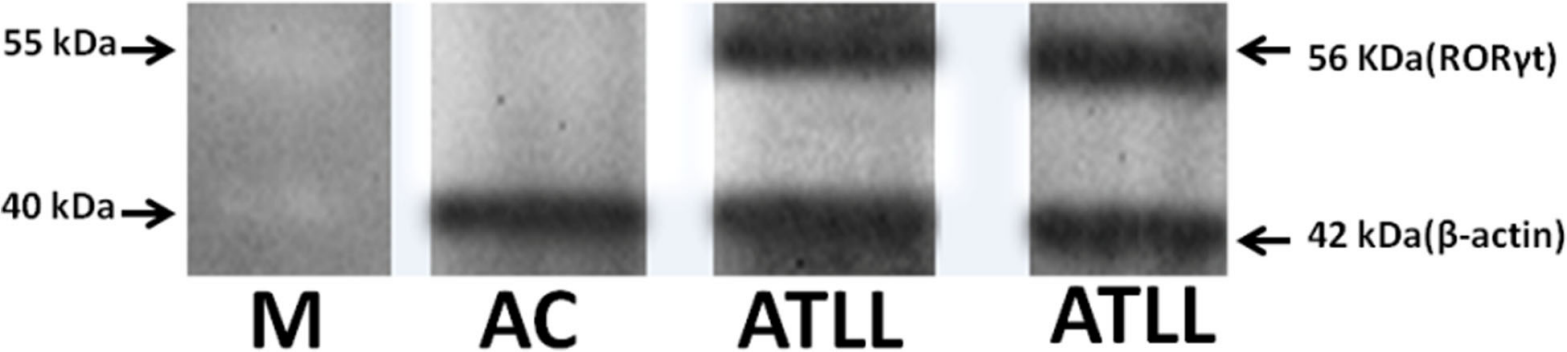
RORγt expression in ATLL and AC subjects. The expression of RORγt (56 kD) and beta actin (42 kD) in equal protein amounts of PBMCs lysate of the subjects were analysed by western blot with anti-RORγt and anti-beta-actin conjugated with horseradish peroxides. RORγt, RAR-related orphan nuclear receptor; ATLL, adult T-cell leukemia/lymphoma; ACs, asympthomatic carriers; M, molecular size marker (kDa)

High-level mRNA expression of *AKT1* and *RORγt* in ATLL patients than ACs was confirmed in protein level by western blotting. Housekeeping Beta-actin gene with 42 kDa molecular weight (MW) was used as reference gene. According to Fig. 8, was observed two protein band for AKT1 with 62 kDa and beta-actin in ATLL patients whereas one protein band for beta-actin in ACs, as well as for RORγt with 56 kDa was seen (Fig. 9).

### Statistical correlation

The correlation between variables was evaluated in the three groups and in each group separately. Significant correlation observed between PVL and *HBZ* mRNA level in ATLL, HAM/TSP and ACs groups (*R* = 0.50, *P* = 0.003). There was a significant association between PVL and IFNλ3 mRNA level in the three groups (*R* = 0.33, *P* = 0.04).

Furthermore, a significant association was observed between *HBZ* and *AKT1* (*R* = 0.37, *P* = 0.03), *HBZ* and *FOXP3* (*R* = 0.44, *P* = 0.03), also between *HBZ* and IFNλ3 mRNA levels (*R* = 0.55, *P* = 0.001,) in the three groups.

In ATLL patients there was a significant association between *AKT1* and *IFNλ3* mRNA expression levels (*R* = 0.55, *P* = 0.02). Moreover, strong significant correlation was observed between *HBZ* and *IFNλ3* mRNA expression levels (*R* = 0.67, *P* = 0.004,) in ACs.

In HAM/TSP patients there was significant association between *AKT1* and *IFNλ3* mRNA expression levels (*R* = 0.97, *P* = 0.003).

## Discussion

It is well known that only 3–5% of infected subjects develop ATLL or HAM/TSP [1], therefore, HTLV-1 infection alone may not be sufficient for developing its associated diseases. Furthermore, over many years after the discovery of HTLV-1, the pathogenicity of this virus in developing ATLL or HAM/TSP in a small proportion of infected individuals, yet to be understood. Therefore, viral–host interactions in context of gene expressions are assumed to be the main effective factors in HTLV-1 associated diseases development and progression. Thus, it is more likely that in addition to viral factors, the host genetic abnormalities and inappropriate epigenetic responses should be considered for understanding of the mechanism of the oncogenesis of ATLL or developing autoimmunity in HAM/TSP.

Although, the previous studies have suggested a possible important role for PVL in the development of ATLL and HAM/TSP, the association between PVL and disease development remains unclear. According to Iwanaga et al. study, the role of high PVL level remains uncertain because the majority of ACs with a high PVL level remained asymptomatic [20]. In the present study, the level of PVL is significantly higher in ATLL patients than in ACs, but the level of PVL is not statistically higher in HAM/TSP patients than in ACs. Of course, the increasing trend of PVL in ATLLs is due to the presence of a huge number of malignant infected cells in ATLL patients. Collectively, in our previous studies and many other authors significant differences in PVL were observed between HTLV-1 associated diseases and ACs [4, 21–23].

In addition to PVL, Tax is produced in the early phases of infection to establish viral replication and induce T cell transformation, proliferation and DNA damage. HBZ is subsequently expressed to inhibit *Tax* expression to escape the host immune pressure for virus elimination by Tax specific CTLs. Therefore, it produces as a second oncogenic signal for the maintenance of the leukemic cells. HBZ is expressed in nearly all malignant cells of the ATLL patients, and its expression is correlated positively with PVLs and negatively with *tax*/*rex* and *gag*/*pol* expression [24]. In the present study, a significant association was also observed between PVL and *HBZ* expression gene since it is possible with an increase of PVL, *HBZ* increment happens in infected cells.

Our findings showed that *HBZ* expression surprisingly showed an 800-fold higher increase in ATLL patients compared with ACs and HAM/TSP patients. Thus, these results are consistent with those of previous studies on the role of *HBZ* in the progression toward leukemogenesis, and support the critical role of *HBZ* in HTLV-1 oncogenesis. In the most cases, *HBZ* is the only HTLV-1 molecule that is expressed and may be involved in the maintenance of most ATLL cells which has an important role in leukemogenesis. Taken together, these studies have suggested that, although *HBZ* has growth-promoting activity in ATLL, it does not participate in transformation [25, 26].

Our findings show no significance difference in *HBZ* expression between HAM/TSP patients and ACs. This finding is consistent with previous studies that were shown more effective role of *Tax* than *HBZ* increment in progression toward HAM/TSP [4, 27].

Apart from these viral factors in the immortalisation of host-infected cells, survival and apoptotic signalling pathways are pivotal in promoting malignancy, such as PI3K pathway and BCL-2 family. The present study focuses on the AKT1 molecule from the PI3K signalling pathway and the pro-apoptotic factor from the intrinsic programmed cell death pathway as an antagonist for BAD. For instance, AKT inactivates BAD through phosphorylation and therefore inactivates its ability to induce apoptosis for promoting cell survival. Conversely, the dephosphorylation of BAD leads to the targeting of BAD to the mitochondrial membrane in which BAD interacts with anti-apoptotic proteins, such as BCL-2 and BCL-xL, to induce apoptosis. Nakahata et al. showed that the PI3K/AKT signalling pathway has a critical role in the induction of ATLL and other malignancies [28]. However, the exact mechanisms by which this signalling pathway is activated in the infected cells of HAM/TSP patients remain unclear [29]. In our study, AKT1 was evaluated as the primary mediator of the PI3K signalling pathway, and the findings showed that *AKT1* expression in the ATLLs is 12–18 times more than the ACs and HAM/TSP patients. These findings showed that the PI3K/AKT1 pathway may have stronger effects on ATLL than on HAM/TSP. In the present study, a significant association was found between *HBZ* and *AKT1* genes expression, thus *HBZ* increment can impact on *AKT1* expression and its subsequent effects on maintenance of HTLV-1 infected cells and ATLL progression. Also, lower *HBZ* gene expression in HAM/TSP patients than ACs may affect low expression of AKT1 in HAM/TSP patients compare with ACs.

Signalling pathways such as NF-κB and PI3K/AKT1 can somehow regulate proteins from the BCL-2 family. Nicot et al. found that the anti-apoptotic *BCL* expression is high and that the level of pro-apoptotic proteins Bax, BAD and Bak are not considerably changed in HTLV-1 infected T cells [30]. In the present study, the expression indices for *BAD* were not significant between ATLL patients and ACs. Conversely, the *AKT1* expression had a significant difference between these groups in Jeong et al. study. Therefore, AKT1 may inhibit the BAD pro-apoptotic molecule and contribute to cell survival [8]. Previous studies have shown that CTL-induced apoptosis of HTLV-1-infected T cells is a possible mechanism for the elimination of HTLV-1-infected cells [31, 32]. Therefore, according to our findings (i.e. a significant difference in *BAD* expression between HAM/TSPs and ACs) and previous studies, *BAD* which involves in cell death, preventing the BAD apoptotic pathway may exacerbate as well as induce the HTLV-1 associated diseases.

Although, ATLL cells display an activated helper/inducer (CD4^+^ and CD25^+^) T-cell phenotype, they are not regarded as having helper functions. Instead, ATLL cells demonstrate a strong immunosuppressive activity in vitro [33]. Kohno et al. cleared that *FOXP3* and/or GITR mRNA is expressed in almost all of the primary ATLL samples [34], and Chen et al. reported the first evidence that ATLL cells have a Treg-like regulatory function [35]. In our study, the *FOXP3* expression level in ATLL cells was significantly higher than that in ACs and HAM/TSP patients. Consistent with this finding Satou et al. showed in HBZ-transgenic mice CD4^+^ CD25^+^ Treg cells increased and also they showed *HBZ* expression directly induced *FOXP3* gene transcription in T cells, however these cells functionally impaired while their proliferation was enhanced, thereby impairing the suppressive function of Treg cells [6].

In the present study, a significant correlation between *HBZ* and *FOXP3* expression were observed, therefore *HBZ* increment could impact on expression and function of *FOXP3*. This finding may indicate that the suppressive activity of Treg is part of the immunodeficiency condition in ATLL patients.

Additionally, no such significant difference was found for *FOXP3* expressions in HAM/TSP patients and ACs. Yamano et al. demonstrated that *FOXP3* expression in HAM/TSP patients was lower than that in CD4^+^CD25^+^ T cells from healthy individuals [36]. Oh et al. also reported HAM/TSP patients have lower of *FOXP3* expression in PBMCs than that in ACs and healthy individuals. Consequently, they suggested that impaired *FOXP3* expression could contribute to the development of inflammatory disease during HTLV-1 infection [37]. The later study is consistent with our result that *FOXP3* expression in inflammatory HAM/TSP reactions remains unchanged. This incapability of their suppressive ability to overcome such a situation results in the progression to inflammatory reactions such as HAM/TSP [38]. Since there was a positive association between *HBZ* and *FOXP3* gene expression therefore, low *HBZ* expression could impact on low *FOXP3* expression in HAM/TSP patients but not ATLL.

It has been demonstrated that the inappropriate production of IL-17 supresses the differentiation of Th1 cells, consequently inhibiting the production of IL-2 and IFN-γ. This suppression results in decrement of CTLs functions in favour of viral dissemination. However, the indirect evidence showed that Th17 sub-population may be necessary for preventing certain viral dissemination. Therefore, the role of Th17 in viral infection can differ depending on the virus, disease stage, and host immune background [18].

In the present study, RORγt was assessed as master regulator of Th17 which had high expression in ATLL patients than HAM/TSP and ACs groups. Moreover, high expression level of *RORγt* mRNA in protein level was confirmed by western blotting. According to what was earlier said about the role of Th17 in viral infections high level of *RORγt* in infected cells through inhibition of Th1 cells may cause HTLV-1 associated diseases. Zhao et al, reported HBZ enhances signalling of TGF-β [39], since TGF-β induce differentiation of Th17 [7, 40] therefore, *HBZ* increment may impact on *RORγt* high-level expression in ATLL patients. Additionally, RORγt expression has increased, consistent with inflammatory reactions in the skin and other ATLL patient’s organs.

The IFNλ3 was considered after observing the presence of specific polymorphisms near the IFNλ3 gene is associated with an increased response to treatment or spontaneous clearance of those that developed chronic hepatitis C [41, 42]. Moreover, Kotenko et al. showed viral elements induce *IFNλ* mRNA in various cell lines [43]. Also, we observed high *IFNλ3* mRNA expression in ATLL and HAM/TSP patients than ACs, however, no significant difference was found between ATLL and HAM/TSP patients. Furthermore, consistent with Kotenko’s study in our study a significant association between viral factors (PVL, *HBZ*) and *IFNλ3* gene expression was found. Besides, significant association between *AKT1* expression and *IFN λ3* was observed expression in both ATLL and HAM/TSP patients, thus this finding indicates high expression of *AKT1* in HTLV-1 infected cell may induce high *IFNλ3* mRNA expression in ATLL and HAM/TSP patients. In the present study, the elevated *IFNλ3* level was observed in HTLV-1 but in infected individuals could not prevent to disease development.

This study has some limitations; the incidence of ATLL even in our endemic region is rare for large sampling. Furthermore, to obtain high and stable RNA extraction for gene expression using quantitative real-time PCR, the PBMCs were treated by TriPure. Therefore, the protein extraction from TriPure for western blot assay did not come to the proper results for all of studied protein, except AKT1 and RORγt.

## Conclusions

Our last bioinformatics study using networking also showed that PI3K/AKT/mTOR, in cell cycle and anti-apoptotic pathways and BCRA-1 as DNA damage repair signalling pathways are main players in ATLL development and progression [44]. The present results, our previous study on LAT, BIM, c-FOS and RAD51 [45] and recent bioinformatics findings demonstrated that in contrast to HAM/TSP patients, AKT/mTOR and DNA repairing pathways such as BRCA1 and RAD51 are pivotal signalling pathways in the maintenance and progression of ATLL which should be targeted for therapy in ATLL malignancy. On the other side, HTLV-1 HBZ should be inhibited to guarantee the therapy and recurrence of the disease. However, more studies are needed to elucidate the additional mechanisms in the small proportion of HTLV-1 infected subjects that progresses to ATLL.

## Abbreviations

ACs: Asymptomatic carriers
AKT1: Serine/threonine kinase 1
ATLL: Adult T-cell leukemia/lymphoma
BAD: Bcl-2-associated death promoter
cDNA: Complementary DNA
CTLs: Cytotoxic T lymphocytes
FOXP3: Forkhead box P3
HAM/TSP: HTLV-1-associated myelopathy/tropical spastic paraparesis
HBZ: HTLV-1 bZIP factor
HTLV-1: Human T-cell leukemia virus type 1
IFN: Interferon
MHC: Major histocompatibility complex
NK-cells: Natural killer cells
PBMCs: Peripheral blood mononuclear cells
PI3K: Phosphoinositide 3-kinase
PVDF: Polyvinylidene fluoride
PVL: Proviral load
Real-time qRT-PCR: Real-time quantitative reverse transcription-polymerase chain reaction.
RORγt: RAR-related orphan nuclear receptor
Tregs: Regulatory T cells
β2M: β2 Microglobulin
